# Commentary: Neuroinflammatory *In Vitro* Cell Culture Models and the Potential Applications for Neurological Disorders

**DOI:** 10.3389/fphar.2021.792614

**Published:** 2021-12-23

**Authors:** Yufang Wang, Yilin Peng, He Yan

**Affiliations:** Department of Forensic Medicine, School of Basic Medical Science, Central South University, Changsha, China

**Keywords:** neuroinflammation, cell lines, BV-2 cell, N9 cell, microglial cells

We read with great interest the article by Peng et al. (2021), “Neuroinflammatory *In Vitro* Cell Culture Models and the Potential Applications for Neurological Disorders,” which appeared in Frontiers in Pharmacology on 23 April 2021. This review was helpful for further understanding the functions and limitations of different cell lines in neuroscience research and inspired our team in selecting *in vitro* cell culture in the neuroinflammatory study.

However, incorrect organismic classification of BV-2 cells was discovered in the body of the article. In the section “Microglia,” the authors mentioned that “The two commonly used cell lines produced by the replacement are BV-2 and N9 cells from rats and mice to quickly produce large numbers of cells, respectively.” Actually, BV-2 and N9 cells were both retroviral immortalized microglia from mice beyond any doubt. Similar errors were found in a review written by Branden et al. (Stansley et al., 2012) in the Journal of Neuroinflammation. In the article, at multiple places, they wrongly claimed that BV-2 cells were used for rat models. Until now, none of the authors have posted explanations and corrected revisions in the journal.

The role of microglia has been a research hotspot in the field of neuroinflammation. With a time-consuming and costly culture process, and low quantities of purified cells from cultivating primary microglia, as an alternative, investigators have created several immortalized cell lines to study the function of microglia *in vitro*, including mice derived BV-2 (Blasi et al., 1990) and N9 cells (Righi et al., 1989), rat derived HAPI cells (Cheepsunthorn et al., 2001), and human derived HMO6 cells (Nagai et al., 2001).

Remarkably, apart from the genomic alterations that have presented them immortal, differences in variance of morphologies, adhesion properties, and proliferation rates have also been observed compared to primary microglial cells. Despite these differences, some researchers continue to use them in experimental culture models under the assumption that they represent primary microglial function to some extent. Among these immortalized cell lines, BV-2 cells derived from newborn brain of inbred C57BL/6 mice by infection with the J2 retrovirus carrying the v-raf/v-myc oncogene have been most extensively used as an *in vitro* culture scheme, including experiments in studying LPS-induced inflammation and classical neurotoxicity, detecting cytokine secretion and cell-surface receptors (e.g., purinergic receptors), examining certain signal molecules and signal pathways (e.g., ERK and MAPK signaling), and even using in electrophysiological studies. Nevertheless, BV-2 cells do not fully express primary microglia characteristics, and some microglia-specific genes were rarely expressed in microglia cell lines (Butovsky et al., 2014). Several comparative studies have revealed differences in molecular signature between these immortalized cell lines and primary microglia (for review, see Henn et al. (2009) and Das et al. (2016)).

Besides BV-2 cells, also other microglia cell lines were available, although they were less frequently used. Most notable were the N9 cells derived from embryonic brain of outbred ICR/CD1 mice by culture with the 3RV retrovirus carrying an activated v-myc/v-mil oncogene of the avian retrovirus MH2, which were habitually employed as parallel control together with BV-2 cells or used in combination with primary microglia to validate the inflammatory response intracellular signaling pathways synergistically. N9 cells shared many phenotypical characteristics with primary microglia, but not to the same extent, including the release of inflammatory cytokines by LPS and the expression of microglial cell surface markers (e.g., positive expression of FcR, Mac-1, and F4/80, negative of GFAP, A2B5, and Gal-C) and receptors (e.g., purinergic receptors) (Stansley et al., 2012).

Although N9 cells have also been shown to be similar to BV-2 cells, a few reports of differences between BV-2 and N9 cells have been published. Collectively, several studies have demonstrated that BV-2 cells have intense phagocytic activity, yet they lack robust secretory activity. In contrast, the N9 cells have been shown to possess the solid secretory activity and produce reactive nitrogen intermediates and cytokines such as TNF-α, IL-6, MCP-1, and IL-8 (Righi et al., 1989; Meda et al., 1995; Zhang et al., 2003; Nikodemova and Watters, 2011). In addition, BV-2 cells were more sensitive to NGF and BDNF than N9 cells (Zhang et al., 2003). Since both neurotrophic factors boost the proliferation and survival of microglia, this result may suggest that BV-2 and N9 cells have diverse potential performances in regulating neuronal cells during CNS repair. Differential immune responses of BV-2 and N9 cells may be due to different immortalization techniques and mice of different genetic backgrounds used to derive these cell lines. There have also been descriptions of other microglia cell lines, but none has undergone characterization similar to BV-2 cells, and their use was quite limited. In general, earlier experiences have taught us that any cell line must first be shown to respond similarly to the primary cells they are to model before they are used in experiments.

Because species of cell lines is a fundamental and non-negligible issue, we wonder whether the mistake was just a slip of the pen. However, the result will not influence the conclusion, which inspired us to pursue a meticulous scientific attitude.

## Abbreviations

BDNF, brain-derived neurotrophic factor; CNS, central nervous system; ERK, extracellular signal–regulated kinase; FcR, receptors for the Fc portion of immunoglobulins; Gal-C, galactocerebroside; GFAP, glial fibrillary acidic protein; IL-6, interleukin 6; IL-8, interleukin 8; LPS, lipopolysaccharide; Mac1, macrophage-1 antigen; MAPK, mitogen-activated protein kinase; MCP-1, monocyte chemoattractant protein-1; NGF, nerve growth factor; TNF-α, tumor necrosis factor alpha.

## References

[B1] Blasi E. Barluzzi R. Bocchini V. Mazzolla R. Bistoni F. (1990). Immortalization of Murine Microglial Cells by a v-raf/v-myc Carrying Retrovirus. J. Neuroimmunol. 27, 229–237. 10.1016/0165-5728(90)90073-v 2110186

[B2] Butovsky O. Jedrychowski M. P. Moore C. S. Cialic R. Lanser A. J. Gabriely G. (2014). Identification of a Unique TGF-β-Dependent Molecular and Functional Signature in Microglia. Nat. Neurosci. 17, 131–143. 10.1038/nn.3599 24316888 PMC4066672

[B3] Cheepsunthorn P. Radov L. Menzies S. Reid J. Connor J. R. (2001). Characterization of a Novel Brain-Derived Microglial Cell Line Isolated from Neonatal Rat Brain. Glia 35, 53–62. 10.1002/glia.1070 11424192

[B4] Das A. Kim S. H. Arifuzzaman S. Yoon T. Chai J. C. Lee Y. S. (2016). Transcriptome Sequencing Reveals that LPS-Triggered Transcriptional Responses in Established Microglia BV2 Cell Lines are Poorly Representative of Primary Microglia. J. Neuroinflamm. 13, 182. 10.1186/s12974-016-0644-1 PMC4940985 27400875

[B5] Henn A. Lund S. Hedtjärn M. Schrattenholz A. Pörzgen P. Leist M. (2009). The Suitability of BV2 Cells as Alternative Model System for Primary Microglia Cultures or for Animal Experiments Examining Brain Inflammation. Altex 26, 83–94. 10.14573/altex.2009.2.83 19565166

[B6] Meda L. Cassatella M. A. Szendrei G. I. Otvos L. Jr. Baron P. Villalba M. (1995). Activation of Microglial Cells by Beta-Amyloid Protein and Interferon-Gamma. Nature 374, 647–650. 10.1038/374647a0 7715705

[B7] Nagai A. Nakagawa E. Hatori K. Choi H. B. McLarnon J. G. Lee M. A. (2001). Generation and Characterization of Immortalized Human Microglial Cell Lines: Expression of Cytokines and Chemokines. Neurobiol. Dis. 8, 1057–1068. 10.1006/nbdi.2001.0437 11741401

[B8] Nikodemova M. Watters J. J. (2011). Outbred ICR/CD1 Mice Display More Severe Neuroinflammation Mediated by Microglial TLR4/CD14 Activation Than Inbred C57Bl/6 Mice. Neuroscience 190, 67–74. 10.1016/j.neuroscience.2011.06.006 21683771 PMC3156380

[B9] Peng Y. Chu S. Yang Y. Zhang Z. Pang Z. Chen N. (2021). Neuroinflammatory *In Vitro* Cell Culture Models and the Potential Applications for Neurological Disorders. Front. Pharmacol. 12, 671734. 10.3389/fphar.2021.671734 33967814 PMC8103160

[B10] Righi M. Mori L. De Libero G. Sironi M. Biondi A. Mantovani A. (1989). Monokine Production by Microglial Cell Clones. Eur. J. Immunol. 19, 1443–1448. 10.1002/eji.1830190815 2789141

[B11] Stansley B. Post J. Hensley K. (2012). A Comparative Review of Cell Culture Systems for the Study of Microglial Biology in Alzheimer’s Disease. J. Neuroinflamm. 9, 115. 10.1186/1742-2094-9-115 PMC3407712 22651808

[B12] Zhang J. Geula C. Lu C. Koziel H. Hatcher L. M. Roisen F. J. (2003). Neurotrophins Regulate Proliferation and Survival of Two Microglial Cell Lines *In Vitro* . Exp. Neurol. 183, 469–481. 10.1016/s0014-4886(03)00222-x 14552887

